# Age-Related Changes in Sleep and Its Implications for Cognitive Decline in Aging Persons With Schizophrenia: A Critical Review

**DOI:** 10.1093/schbul/sbae059

**Published:** 2024-05-07

**Authors:** Bengi Baran, Ellen E Lee

**Affiliations:** Department of Psychological and Brain Sciences, University of Iowa, Iowa City, IA, USA; Department of Psychiatry, Carver College of Medicine, University of Iowa, Iowa City, IA, USA; Iowa Neuroscience Institute, Carver College of Medicine, University of Iowa, Iowa City, IA, USA; Department of Psychiatry, University of California San Diego, La Jolla, CA, USA; Desert-Pacific Mental Illness Research Education and Clinical Center, Veterans Affairs San Diego Healthcare System, San Diego, CA, USA

**Keywords:** non-rapid eye movement sleep/, aging/, obstructive sleep apnea/, cognitive deficits

## Abstract

**Background and Hypothesis:**

Cognitive impairment is a core feature of schizophrenia that worsens with aging and interferes with quality of life. Recent work identifies sleep as an actionable target to alleviate cognitive deficits. Cardinal non-rapid eye movement (NREM) sleep oscillations such as sleep spindles and slow oscillations are critical for cognition. People living with schizophrenia (PLWS) and their first-degree relatives have a specific reduction in sleep spindles and an abnormality in their temporal coordination with slow oscillations that predict impaired memory consolidation. While NREM oscillatory activity is reduced in typical aging, it is not known how further disruption in these oscillations contributes to cognitive decline in older PLWS. Another understudied risk factor for cognitive deficits among older PLWS is obstructive sleep apnea (OSA) which may contribute to cognitive decline.

**Study Design:**

We conducted a narrative review to examine the published literature on aging, OSA, and NREM sleep oscillations in PLWS.

**Study Results:**

Spindles are propagated via thalamocortical feedback loops, and this circuitry shows abnormal hyperconnectivity in schizophrenia as revealed by structural and functional MRI studies. While the risk and severity of OSA increase with age, older PLWS are particularly vulnerable to OSA-related cognitive deficits because OSA is often underdiagnosed and undertreated, and OSA adds further damage to the circuitry that generates NREM sleep oscillations.

**Conclusions:**

We highlight the critical need to study NREM sleep in older PWLS and propose that identifying and treating OSA in older PLWS will provide an avenue to potentially mitigate and prevent cognitive decline.

## Introduction

Older people living with schizophrenia (PLWS) are a rapidly growing population^1^ with high-risk of cognitive impairment. Cognitive impairment is a highly disabling core feature of schizophrenia^2^ that interferes with quality of life, daily functioning, and independence.^3^ Impaired functioning and disability are responsible for the majority of the economic burden associated with schizophrenia, which has more than doubled since 2013.^4,5^ Cognitive deficits are reported to begin in the prodromal phase of the illness^6^ and worsen with aging, exposure to medication, and unhealthy lifestyle factors.^7,8^ Currently available treatments, including antipsychotic drugs^9,10^ result in minimal change in cognitive deficits, highlighting the need for novel treatment targets. The goals of this review are to (1) briefly summarize the cognitive function of sleep, (2) identify the specific sleep deficit in schizophrenia that interferes with cognition, (3) discuss age-related changes in sleep and their implications for cognition, (4) explain that obstructive sleep apnea, when untreated, exacerbates age-related cognitive decline, (5) point to the critical need to investigate the additive effects of aging and obstructive sleep apnea on sleep-mediated cognitive deficits in schizophrenia (see Table 1 for a summary of key studies).

**Table 1.** Key Studies on Sleep in Schizophrenia Highlighted In This Review and Their Main Findings:

Citation	Sample	Mean Age	Methods	Summary of Findings
Kozhemiako et al^11^	72 PLWS 58 controls	PLWS: 35 ± 7 y Controls: 32 ± 6 y	High-density nocturnal EEG to characterize NREM sleep parameters	A large-sample study that replicates previous findings of reductions in sleep spindle density and morphology in schizophrenia, reduced spectral power in 2–6 Hz and 12–15 Hz bands, and altered slow oscillation characteristics; provides novel findings with respect to altered intra spindle frequency deceleration in schizophrenia.
Baran et al^12^	22 PLWS 29 controls	PLWS: 32 ± 7 y Controls: 30 ± 6 y	High-density nocturnal EEG to characterize sleep spindles. Resting-state functional MRI in a separate session to characterize thalamocortical connectivity	Increased thalamic connectivity with bilateral somatosensory and motor cortex and reduced sleep spindle density in PLWS; thalamic hyperconnectivity with the sensorimotor cortex correlated with reduced spindle density.
Buchmann et al^13^	21 PLWS 21 controls	PLWS: 36 ± 8 y Controls: 36 ± 10 y	High-density nocturnal EEG to characterize sleep spindles. Structural MRI in a separate session to characterize thalamic volumetry	Reduced volume of the bilateral mediodorsal nucleus of the thalamus in PLWS; medidorsal thalamic volume correlated positively with sleep spindle density.
Ancoli-Israel et al^14^	52 PLWS	60 ± 5 y	Nocturnal sleep monitoring with pulse oximetry, respiration, and EMG	High prevalence of sleep-disordered breathing: 48% of PLWS had at least 10 respiratory events per hour of sleep; lower Mini-Mental State Exam scores among individuals with sleep-disordered breathing.
Szaulińska et al^15^	51 PLWS 31 PLWS and obesity 51 controls (18 with obesity)	PLWS: 38 ± 12 y PLWS and obesity: 39 ± 12 y Controls: 38 ± 12 y	OSA risk assessed with STOP-BANG, NoSAS, no-apnea, and Berlin Questionnaires	More severe negative symptoms, more daytime sleepiness and reduced digit-symbol test performance in PLWS with OSA.
Myles et al^16^	6 PLWS with severe untreated OSA who accepted CPAP treatment	37 ± 12 y	Six months of CPAP treatment, baseline and follow-up assessments with sleep EEG and cognitive testing	CPAP treatment in schizophrenia is associated with improvements in cognition, weight loss, and increased time spend in slow wave and REM sleep.

As reviewed extensively by others,^17–20^ sleep serves a crucial cognitive function: consolidation of new learning. Demonstrated with carefully controlled experiments, this “sleep benefit” is not merely a passive protection from interference.^21^ Rather, temporal coordination of 3 cardinal non-rapid eye movement (NREM) sleep oscillations create the ideal milieu for the transfer of new memory traces from the hippocampus to more stable representations in the cortex^22^: Sleep spindles are waxing and waning ~12-15 Hz oscillations initiated by the thalamic reticular nucleus and propagated to the cortex by thalamocortical circuitry; slow oscillations (SO) are highly synchronous ~0.5-1.25 Hz oscillations of prefrontal cortical origin; and sharp wave ripples are ~80-150 Hz oscillations originating from the hippocampus. In rodent models, optogenetic induction of sleep spindles in the rising upstate of the SO increases co-occurrence of these 3 oscillations and enhances hippocampal memory consolidation whereas inhibiting thalamic reticular neurons and, thus preventing spindles from phase-locking with these oscillations impairs memory.^23^ While it may not be possible to detect hippocampal ripples with traditional surface EEG acquisition and analysis methods, intracranial recordings in presurgical epilepsy patients confirm the finding that sleep spindles mediate the dialogue between the cortex and the hippocampus.^24^ Beyond their role in sleep-dependent memory consolidation, hippocampal sharp wave ripples have recently been shown to modulate blood glucose levels, suggesting that deficits in hippocampal ripples may play a role in metabolic dysfunction.^25^

Several lines of evidence identify a causal role for NREM oscillations in memory consolidation. Spindles predict sleep-dependent changes in memory performance for both procedural and declarative memory,^17^ increasing spindles through transcranial electrical stimulation enhances sleep-dependent memory consolidation,^26^ and learning a new task corresponds to localized increases in spindle activity during subsequent sleep in cortical regions involved in task performance.^27–29^ Beyond memory consolidation, spindles correlate with general cognitive abilities as indexed by IQ or neuropsychological assessments of learning, attention, working memory, and verbal fluency.^30,31^

## Sleep Spindles in Schizophrenia

People living with schizophrenia have a specific reduction in sleep spindles that correlates with deficits in sleep-dependent memory consolidation.^32,33^ In-lab polysomnography studies in chronic, medicated PLWS reveal that these oscillatory deficits occur in the context of sleep architecture and EEG power in other bands comparable to matched controls.^11,34,35^ Importantly, spindle deficits are not due to disease chronicity or medication side effects. Antipsychotic-naïve, early-course patients with schizophrenia^36^ and individuals at clinical high-risk for psychosis^37^ exhibit a significant reduction in spindle activity that correlates with performance on tests of executive function, working memory and IQ. Spindle deficits are not related to type or dosage of antipsychotic medication.^38^ Further, sleep spindles are also reduced in young, non-psychotic first-degree relatives of schizophrenia.^36,39,40^ This evidence identify sleep spindle deficits as a biomarker of schizophrenia that reflects genetic vulnerability and contributes to cognitive deficits.^41^

Sleep spindles are initiated by the thalamic reticular nucleus and propagated by thalamocortical feedback loops. Quantified with resting-state functional connectivity MRI and diffusion tensor imaging, structural and functional connectivity of thalamocortical circuitry is abnormal in scizhophrenia^42–44^: connectivity between the thalamus and primary motor and sensory regions is abnormally increased in PLWS. Spindle deficits in PLWS are related to this hyperconnectivity pattern, such that reduced spindle density correlates with increased sensorimotor thalamocortical connectivity.^12^ Beyond its connectivity with the cortex, structural abnormalities of the thalamus have also been identified in PLWS, such that reduced volume of the mediodorsal nucleus correlates with sleep spindle density.^13^

Pharmacologically increasing sleep spindles in PLWS with eszopiclone, a non-benzodiazepine hypnotic, fails to improve sleep-dependent memory consolidation, presumably because drug-induced increases in spindles fail to coordinate with other NREM oscillations.^34^ Other NREM oscillatory deficits observed in PLWS are reductions in slow-wave activity^45,46^ and alterations in the density and morphology of SO^11^ (also see^38^). Importantly, the temporal coordination of SOs with spindles is abnormal, and SO-spindle coordination and spindle density together explain memory consolidation deficits better than just spindles alone.^47^ In addition to cognitive deficits, NREM oscillation abnormalities in PLWS have been shown to correlate with the severity of positive^48,49^ and negative symptoms.^50^ Postmortem studies, human neuroimaging and animal models all converge on the finding that schizophrenia is associated with a loss of parvalbumin-positive interneurons in the hippocampus.^51^ While there is no study that has examined NREM hippocampal sharp wave ripples in PLWS, it has been speculated that the loss of these GABAergic interneurons leads to a reduction in ripples and contribute to sleep-dependent memory consolidation deficits.^52^ Taken together, this body of work identifies NREM oscillations as a biomarker for schizophrenia and the circuitry that propagates NREM oscillations as a target for alleviating cognitive deficits and symptoms.

## Obstructive Sleep Apnea in Schizophrenia

In addition to high rates of insomnia and circadian disturbances,^53^ nearly 46–70% of PLWS meet the criteria for OSA,^14,54–58^ compared with 9–38% of the general population.^59^ Elevated OSA risk among PLWS appears to be linked to obesity, comorbid metabolic and cardiovascular disease, psychopathology, and antipsychotic medication usage.^60–62^ However, OSA among PLWS is often underdiagnosed. In fact, a Veterans Affairs medical records study estimated that only 2% of persons with psychotic disorders had formal diagnoses of OSA.^63^ Moreover, commonly used OSA risk scales may be less accurate among PLWS. For example, the specificity of the STOP-BANG OSA risk rating questionnaire, NoSAS OSA risk rating questionnaire, Berlin Questionnaire, and NoApnea OSA risk rating questionnaire was quite low at 0.53–0.64.^15^ The high rates of false positives were attributed to common complaints of fatigue, low energy, and overnight awakenings among people with schizophrenia. Also, many PLWS do not have bed partners to contribute information regarding snoring or pauses in breathing overnight. Szaulinksa et al. found that simply using obesity (BMI ≥ 30kg/m^2^) and large neck circumference (≥ 41cm in women, ≥ 43cm in men) had better specificity (0.77), thus with fewer false positives.^15^ Beyond risk questionnaires, few studies have systematically screened community-dwelling samples of older PLWS for OSA using objective clinical-grade assessments, such as polysomnography or home sleep tests.

Similarly, few studies have examined the associations of OSA with cognitive functioning among PLWS. One study of 82 PLWS in an inpatient psychiatric unit found that individuals with an OSA diagnosis (Apnea Hypopnea Index ≥ 5 events/hour) had worse performance on the Digit-Symbol test (*p*< .05, *d* = −0.46).^15^ One study of 52 community-dwelling PLWS found lower Mini-Mental State Exam scores (*d* = −0.68) among individuals with sleep-disordered breathing (Respiratory Disturbance Index > 10).^14^ Neither study used standard neuropsychological batteries that are more sensitive to domain-specific deficits. Both studies were cross-sectional in design and could not establish the causal relationships between OSA and cognitive deficits nor account for potential confounders including obesity, medications, and physical comorbidities. Studies have shown that cardiovascular disease and metabolic dysfunction independently lead to cognitive deficits in the general population^64–66^ and among PLWS.^67,68^ Thus, there is a need for longitudinal studies and intervention trials as well as studies that consider the influence of comorbidities that also affect cognitive functioning.

Within the general population, OSA treatment using continuous positive airway pressure (CPAP) therapy can improve cognitive outcomes for subgroups of OSA patients—depending on age, OSA severity, sleepiness, and specific treatment modality.^69–73^ To our knowledge, there is only one published pilot study of CPAP treatment in 6 PLWS which reported that CPAP treatment improved overall cognitive functioning.^16^ Effectiveness of PAP treatment is impacted by adherence to the nightly use of the CPAP machines, which is suboptimal among the general population, ranging from 40% to -85% (defined as using device ≥ 4 h per night for ≥ 70% of the nights, though longer duration/more frequent usage is associated with further benefits).^74^ Due to higher rates of medication nonadherence, psychiatric symptoms, limited access to preventative care, and physical comorbidities among PLWS, CPAP adherence poses a likely obstacle to achieving the benefits of OSA treatment. However, a recent case-control study from an Australian Sleep Medicine Clinic found that individuals with psychotic disorders had similar CPAP adherence at the end of a 3–6 week trial, compared with a non-psychiatric comparison group with OSA (5.7 h/night compared with 4.8 h/night, respectively).^75^ The clinic had standardized procedures to increase adherence for CPAP trials, including individual mask-fitting and weekly appointments during the home CPAP trial. These findings were mirrored by a case-control study of veterans with schizophrenia who had similar PAP usage (% days of uses for > 4 h) at 1- and 3-year follow-up, compared with veterans with OSA and no psychiatric comorbidities (36% vs 49%, and 42% vs 61%, respectively.)^76^ These adherence rates may reflect the relative stability of the cohorts in these studies, PLWS whose psychiatric symptoms are well-controlled and/or have social support such that they are able to follow-up with the sleep medicine specialists to garner a diagnosis of OSA and engage in treatment. Work in other populations found improved CPAP adherence with cognitive behavioral therapy.^77^ Furthermore, with similar adherence rates, the group with psychosis had similar responses to CPAP treatment with post-CPAP reductions in AHI and daytime sleepiness that were comparable to a non-psychiatric comparison group with OSA.^75^

While other OSA treatments (oral appliance therapies, hypoglossal nerve stimulation, surgical interventions, medications, myofunctional therapies)^78^ show promise in other populations, we found very few published studies of these interventions among PLWS. One study of oral appliance therapy treatment for patients with and without psychiatric comorbidities (4 of the 106 participants had schizophrenia) found 2-fold higher rates of treatment discontinuation among the psychiatric comorbidity group.^79^ However, the study showed improvements in AHI and daytime sleepiness among patients with psychiatric comorbidities who continued the treatment. Similarly, several studies of hypoglossal nerve stimulation for patients with Down Syndrome and OSA show promise in improving OSA, as well as cognitive, and behavioral outcomes.^80,81^ Of note, non-CPAP interventions may be less accessible to PLWS, due to lower rates of private insurance^82^ and frequent transitions in health insurance.^83^ One recent review found that individuals who received surgery and oral appliance therapies were often younger (< 40 years old), not obese (BMI ≤ 30 Kg/m^2^), with private insurance and higher income levels.^84^ For hypoglossal nerve stimulation, active psychiatric disease and physical comorbidities are current exclusion criteria.^85^ On the other hand, CPAP treatment is generally covered by public insurance plans. The Australian CPAP trial noted that the participants with psychosis were more likely to rely on government assistance and disability funding to acquire CPAP machines for long-term use compared to the non-psychiatric comparison group.^75^ Barriers to PAP treatment may include lower access to primary care and referrals, stigma, as well as limited social supports.^86,87^ Further work on access to, adherence to, and effectiveness of PAP and other OSA treatments among PLWS is warranted. Specialized adherence protocols may be critical for PLWS to fully benefit from OSA treatments.

## Age-Related Changes in Sleep and Implications for Cognition

Normal aging is associated with marked changes in sleep quality, duration, and architecture. Compared with young adults, older adults have shorter total sleep duration, reduced time in NREM Stages 2 and 3 sleep, increased sleep onset latency and wake after sleep onset, and an increased number of arousals.^88,89^ Sleep fragmentation has also been associated with exaggerated age-related cognitive decline and increased risk of developing Alzheimer’s disease.^90^ Further, a pooled longitudinal cohort study revealed an inverted U-shaped pattern of the relations between sleep duration and cognition, suggesting increased age-related cognitive decline in older adults with both insufficient and excessive sleep.^91^

Parallel to these changes in sleep macrostructure, the occurrence, morphological characteristics, and orchestration of NREM oscillations are also altered with aging. Independent from duration of NREM, the density, amplitude, and duration of sleep spindles are reduced in older adults.^92,93^ Reduced sleep spindle density in aging has been linked to impaired memory as well as reduced hippocampal activation.^94^ Similarly, slow-wave activity as well as density, peak-to-peak amplitude, slope and duration of SO decrease with aging^95,96^ While changes in slow waves are mechanistically related to age-related reductions in homeostatic sleep drive (e.g.^97^), reduced slow-wave activity predicts memory deficits in aging.^98^ In fact, sleep-dependent memory consolidation is reduced in aging,^99,100^ and changes in NREM sleep correlate with altered activation and task-dependent connectivity during memory recall after sleep.^101^

## Obstructive Sleep Apnea in Aging

Older age is also associated with increased OSA risk, with a recent meta-analysis estimating that 35.9% of older adults (65+) have OSA.^102^ OSA diagnosis and severity have been associated with obesity, alcohol consumption, cardiovascular disease, diabetes, hypertension, and metabolic syndrome.^103^ Moreover, OSA severity and low oxygen saturation have been associated with aging biology: C-reactive protein levels, insulin resistance, mitochondrial dysfunction, and genomic instability.^104^ Sleep-disordered breathing in older adults increases the likelihood of developing cognitive impairment.^105^ Several studies have also identified gray matter alterations in older adults with OSA.^106,107^ While the majority of the brain imaging studies point to a volume loss in OSA, sex-specific analyses reveal increased hippocampal volume in older females with OSA that is attributed to edema (extracellular free water accumulation).^108^

Obstructive sleep apnea also interferes with NREM oscillations. Previous work reveals reduced spectral power in the spindle range,^109^ reduced spindle frequency,^110^ reduced density of fast spindles (13–16 Hz),^111^ and abnormal spindle deceleration^112^ in OSA. In older adults with mild cognitive impairment, deficits in sleep-dependent memory consolidation correlate with the severity of apnea/hypopnea events.^113^ Sleep-dependent consolidation of emotional^114^ and procedural memory^114,115^ is significantly reduced in individuals with OSA. Further, thalamic connectivity with sensorimotor regions is increased in OSA^116^ compared to matched controls.

## Why Older People With Schizophrenia Are Particularly Vulnerable to Sleep-Related Cognitive Impairments

As reviewed above, there is a significant overlap between the effects of OSA and schizophrenia on NREM oscillations, the integrity of the brain circuitry that generates these oscillations, and consequently, sleep-dependent memory consolidation. While cognitive deficits (on average 1 SD below the mean for age-comparable individuals)^6^ are present during the prodromal phase and illness onset, these deficits appear to be stable during adulthood^117–119^ but with older age PLWS are more likely to exhibit dementia-level impairments. One recent 2021 study of Medicare beneficiaries (age 66+) reported that PLWS had a 10- to 20-fold higher prevalence of dementia, compared to the general population, with steep age-related increases.^120^ It could be speculated that this reflects accelerated brain aging. In support of this hypothesis, brain age gap calculations (ie, the difference between neuroanatomical and chronological age based on magnetic resonance imaging volumetry analyses) have revealed an average estimate of 2.5–5.5 years of an age gap in PLWS.^121,122^ Further, a recent, large-sample, case-control study of more than 3500 middle-aged individuals compared DNA methylation patterns between psychosis and control groups and revealed accelerated epigenetic aging in patients that was independent of tobacco use or clozapine dosage.^123^

With the rapidly growing older population with schizophrenia and the steep increases in health-related costs, older PLWS are a high-risk and vulnerable group for cognitive impairment with critical need for targeted treatments. Obstructive sleep apnea is an important modifiable risk factor that warrants further investigation into the underlying biological mechanisms. Furthermore, focused diagnosis and treatment of primary sleep disorders among PLWS are crucial to improving overall health and clinical outcomes for this population. Diminishing structural barriers to care and building supportive structures to bolster adherence will be necessary future steps to translate research findings into clinical care.

## Future Research Agenda

The lack of interventions to improve cognition among older PLWS reflects that age-related cognitive decline in schizophrenia is poorly understood. This highlights the need for future studies that investigate causal links between age-related changes in cognition and sleep disruption in schizophrenia. Future work should elucidate the effects of OSA and aging on NREM oscillations in schizophrenia and test whether OSA has an independent additive contribution to age-related cognitive decline in PLWS. Sleep spindle deficits and other proximal brain changes may serve as key biomarkers of cognitive deficits and of response to sleep therapies, such as CPAP treatment. Clarifying specific neurobiological mechanisms underlying sleep-related cognitive outcomes will enable researchers to identify brain-based targets to develop novel interventions such as closed-loop transcranial direct current stimulation (tDCS) or auditory stimulation to enhance NREM oscillations. Such approaches may be instrumental in improving outcomes among middle-aged and older PLWS, a highly vulnerable and understudied population.
